# Immune-molecular interactions in high-grade serous ovarian cancer distinguish long-term survivors

**DOI:** 10.1172/JCI184790

**Published:** 2024-12-16

**Authors:** Jeanette E. Boudreau

**Affiliations:** 1 Department of Microbiology and Immunology,; 2 Department of Pathology, Dalhousie University, Halifax, Nova Scotia, Canada.; 3 Beatrice Hunter Cancer Research Institute, Halifax, Nova Scotia, Canada.

## Abstract

The approach and efficacy of treatments for high-grade serous carcinoma (HGSC) of the ovary have changed little in decades. Although numerous studies demonstrated immune infiltration as frequent and prognostically beneficial, clinical trials of immunotherapies have generated benefit in fewer than 15% of patients. In this issue of the *JCI*, Nelson and colleagues compiled 1,233 HGSC samples from patients across four continents and compared the molecular and immunologic features that associate with long-term survival (greater than 10 years). Diversity among HGSC tumors is well defined, but this study explored the combined influence of immunologic and molecular features. Long-term survivors harbored tumors with high epithelial content and overrepresentation of the C4/differentiated molecular signature, with cytotoxic T and B cells infiltrating to the tumor epithelium and stroma, respectively. These findings highlight features that might underly poor responsiveness to existing immunotherapies of most HGSC tumors and considerations for the design of future, more precise treatments for HGSC.

## HGSC’s diversity demands a precise approach to treatment

High-grade serous carcinoma (HGSC) of the ovary is the most common type of ovarian cancer, accounting for approximately 60% of all cases (1). HGSC is the deadliest gynecologic malignancy, having five-year survival rates of approximately 40% (2, 3). For patients, surgery is usually the first step in a treatment regime, with monitoring and cycles of remissions punctuated by relapses, platinum-based chemotherapy, and further pharmacologic intervention (1). HGSC has been relatively refractory to existing immunotherapies: fewer than 20 % of patients exhibited at least a partial response to anti–PD-L1 in clinical trials (4, 5). The progression of HGSC differs between patients. In some, the cancer is refractory to standard platinum-based chemotherapy and progresses rapidly. In others, survival is intermediate (i.e., 5–8 years) or progression does not occur for 10 years or more (approximately 16%). In this issue of the *JCI*, Nelson et al. (6). reasoned that features of the long-term survivor group might inform HGSC prognostication and treatment design.

HGSC is diverse in its composition of tumor epithelium and stromal compartments, immune infiltrate, and molecular features, including gene expression, ploidy, and mutational burden (7–9). Even within the same patient, individual tumor sites or patches in tumors can have different features (8, 10). Perhaps this diversity reflects tumor evolution over time and under pressure from its interaction with immune cells and chemotherapy, or distinct molecular features that are selected as the tumors progress. While numerous studies have demonstrated that both molecular classifications and immunologic features can predict survival, a study to understand their intersection has been out of reach in single-cohort investigations.

Understanding the concurrent immune and molecular contexture of patients among long-term survivors requires a highly collaborative effort because these patients are rare. The Multidisciplinary Ovarian Cancer Outcomes Group (MOCOG) combines the sample cohorts and efforts of 19 studies across four continents. This collaboration enabled assembly of a large and balanced group of patients based on their lengths of progression-free survival: Nelson et al. compared patients with short (2–5 year), intermediate (5–8 year), and long-term (10 year +) survival. With 1,233 MOCOG samples, Nelson et al. (6) validated, extended, and defined features previously reported to associate with prognostic benefit (6). In a commendable contribution toward the understanding of HGSC, the authors generated comparable and internally controlled data from samples were collected over a 32-year period, at different centers, and undoubtedly with nonidentical protocols.

## Immunologic and molecular features of HGSC

Independent studies have demonstrated that survival in patients is positively correlated with the extent of infiltration of T cells, macrophages, B cells, and natural killer cells (11–13). More recently, and with spatial biology approaches, it is becoming clear that the coinfiltration, localization, and organization of immune cell subsets are especially beneficial (11, 13, 14), suggesting that cooperation between immune cells and how they interact with tumor features could be key to HGSC control. In the study by Nelson et. al. (6), the prognostic benefit was further underscored: infiltration of B and T cells to the stromal and epithelial regions, respectively, were particularly prognostically beneficial in long-term survivors (6). These observations imply the presence of an ongoing antitumor response in the tumor microenvironment that may contribute to tumor control even after surgical debulking.

Most HGSC tumors harbor mutations in the *TP53* gene, which contributes to instability that enables the disease’s diversity (15). Further somatic mutations, copy number alterations, and epigenetic modifications contribute to tumor diversity, with effects on tumor progression and sensitivity to chemotherapy and poly-ADP ribose polymerase (PARP) inhibitors (15–17). Studies of immune cell infiltration and its influence in HGSC are almost always conducted on treatment-naive samples from upfront debulking surgeries, after which patients would proceed to the standard of care: platinum-based chemotherapy. Features of a patient’s cancer seem to be imprinted, with patients having similar molecular signatures at primary and metastatic sites (17), with pressure from the immune system driving clonal diversification (10), so understanding molecular-immune interactions could unveil opportunities for precision medicine. Studies on treatment-naive samples — like the present study by Nelson et al. (6) — likely give insight to an immunologic “setpoint” from which a patient will respond to subsequent treatments. Little is known about the effect of chemotherapy on the distribution and activity of immune cells, but emerging work suggests maintenance, expansion, diversification, and redistribution of cytotoxic T cells, alongside recruitment of additional immune effectors (18). The anticancer immune response can be temporarily suppressed by chemotherapy, but T cell exhaustion may be rescued by immune checkpoint blockade (18), or the genetic features of a tumour might help to predict the features of an existing antitumor immune response; understanding these perspectives might inform how and when to apply emerging and existing treatments, including checkpoint immunotherapy.

Transcriptome assessments enable division of HGSC into four molecular subtypes that differ with respect to their immune infiltration and prognosis for patients with defining features (8). The C2 “immunoreactive” and C4 “differentiated” subtypes associate with the longest survival, and C1 “mesenchymal” associates with the shortest survival interval. A fourth subtype, C5 “proliferative”, exhibits intermediate survival (8). Immunologically, these transcriptomic classifications indicate that the C2 subtype is most heavily infiltrated with immune cells, and C4 corresponds with only intermediate immune cell infiltration (8). Expanding beyond molecular analysis, Nelson et al. demonstrated that long-term survivors are characterized as having immune-infiltrated C4 tumors (6), demonstrating that there is a synergy between the tumor subtype and its interaction with the immune system. C4 tumors contained the highest epithelial content and were the most differentiated. C4 tumors thus likely represent a molecular configuration best targeted by the immune system, or one that seeds few suppressive cells that are associated with the HGSC stroma.

## Molecular and immunologic features in cancer progression

Immune and molecular classifications are prognostically informative, but they represent an average; the composition of HGSC tumors is a mosaic. Within and between tumors, there can be regions relatively devoid of immune cells, stroma-to-epithelium area ratios can differ, and metabolic features can shift (10, 13, 19). All tumors are relatively advanced in a cohort of patients with HGSC (most are diagnosed at stage III or IV).

**Figure 1:**
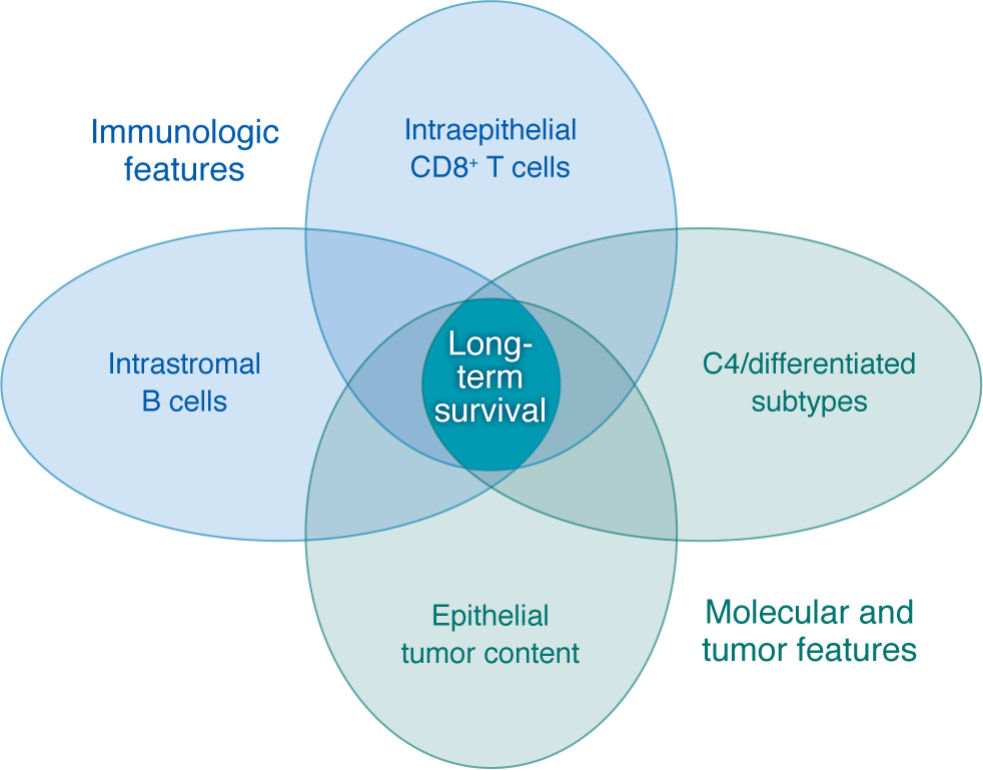
Immunologic and molecular features predict long-term survival. Long term survival among patients with HGSC was best predicted by the coexistence of high-epithelial content tumors, C4 molecular subtype features, intraepithelial CD8^+^ T cells, and intrastromal B cells. While each of these features has been associated with HGSC outcomes previously, this is the first study to demonstrate their intersection. Intriguingly, the association of immune cell subsets was predictive of long term survival only in the context of the C4 molecular subtype, which is not itself classified as typically having a high immune cell infiltration.

Nelson et al. (6) demonstrated that the presence of immune cells was a constant feature among long-term survivors. In those whose tumors classified as the C4 differentiated molecular subtype, stromal B cells and epithelial CD8^+^ T cells were overrepresented. Independently, these immune cell features would not have predicted the best outcomes, but in the context of cancer subtype, it becomes clear that the organization of immune cells in C4 subtype tumors predict long-term survival (Figure 1). C4 subtype tumors exhibit markedly higher epithelial content, higher differentiation, fewer subclones, and lower ploidy, which makes them more similar to low-grade serous tumors (20). This diversity could enable escape from immunologic or pharmaceutical pressure and subsequent tumor evolution and recurrence. C4 tumors are not typically the most immune infiltrated, but perhaps the quality of their anticancer response is superior to that in other tumor subtypes.

Productive immune responses require cooperation between immunologic subsets. For example, plasma B cells release antibodies to induce complement activation or antibody-dependent cellular cytotoxicity or phagocytosis by other leukocytes. T cells aid in activation of B cells, and activation of antigen-specific antitumor immune responses depends on release and presentation of antigen. The Nelson et al. study demonstrates that effector T cells localize to epithelial regions (6); here, they may contribute to direct tumor cell killing. B cells, in contrast, are most prognostically informative localized to the stromal region (6); their role may, therefore, be to produce soluble mediators like antibodies that act from a distance. With time and treatment, tumors may progress away from productive immunity and eventually lead to relapse and death, but long-term survivors seem to start with better immune reactivity. Indeed, studies following cohorts of patients reveal loss of immune signaling and infiltration as the disease progresses and immunologically “cold” tumors at the time of death (19, 21, 22).

## Implications and open questions

Studies of immune-HGSC interactions hope to identify opportunities for therapeutic intervention and immunotherapy design. Although immunotherapy has been unsuccessful in HGSC cohort studies, a small fraction of patients do respond; perhaps these patients have particular molecular and/or immunologic features that could be used to identify them early during their treatment. Patients with a predominant C4 molecular subtype with T and B cell infiltration would be candidates, as they may have a preexisting antitumor response that could be rescued by checkpoint blockade. In the remaining majority of patients, additional or combined interventions might be needed in order to first establish antitumor immunity or enable immune infiltration to tumors. Though the specific antigen(s) targeted by T and B cells were not investigated in Nelson et al. (6), it is intriguing that the best cancer control occurred in tumors that would not likely carry the highest neoantigen burden. Initiation of a productive immune niche could ostensibly enable epitope spreading and activation of further anticancer immune responses, underscoring the requirement for a cooperative immune response.

Whether the molecular features of the tumor promote a particular immune configuration or whether it is the ongoing immune-tumor interaction that promotes evolution of a tumor’s molecular features remain open questions. The Nelson et al. (6) study is observational; C4 molecular features and immune configurations could evolve over time. Understanding their relationship to therapy and response will be important for designing the next generation of immunotherapies and precise definition of treatment programs for patients. By linking molecular features with immunologic ones and highlighting the ideal configuration to promote long-term survival, Nelson and colleagues have set a goalpost for tumor immunity and demonstrated that molecular features may be employed in patient treatment stratification.

## Version 1 12/16/2024

Electronic publication
